# X-Linked Myotubular Myopathy in a Female Patient with a Pathogenic Variant in the *MTM1* Gene

**DOI:** 10.3390/ijms24098409

**Published:** 2023-05-07

**Authors:** Polina Chausova, Aysylu Murtazina, Anna Stepanova, Artem Borovicov, Valeriia Kovalskaia, Nina Ryadninskaya, Alena Chukhrova, Oxana Ryzhkova, Aleksander Poliakov

**Affiliations:** Research Centre for Medical Genetics, Moskvorechie Str. 1, 115522 Moscow, Russia; polinaalex85@gmail.com (P.C.); aysylumurtazina@gmail.com (A.M.); cany@yandex.ru (A.S.); borovikov33@gmail.com (A.B.); mikhailova.v.a@mail.ru (V.K.); outremal@yandex.ru (N.R.); achukhrova@yandex.ru (A.C.); ryzhkova@dnalab.ru (O.R.)

**Keywords:** X-linked centronuclear myopathy, *MTM1*, congenital myopathy

## Abstract

X-linked centronuclear myopathy is caused by pathogenic variants in the *MTM1* gene, which encodes myotubularin, a phosphatidylinositol 3-phosphate (PI3P) phosphatase. This form of congenital myopathy predominantly affects males. This study presents a case of X-linked myotubular myopathy in a female carrier of a pathogenic c.1261-10A>G variant in the *MTM1* gene.

## 1. Introduction

Centronuclear myopathy (CNM) is a genetically heterogeneous group of neuromuscular disorders characterized by muscular weakness and hypotonia. Histochemical analysis of muscular tissue biopsy shows abnormally positioned nuclei in skeletal muscle cells. Centronuclear myopathies are subdivided into three forms: X-linked CNM or myotubular myopathy 1 (caused by variants in the *MTM1* gene), autosomal-dominant CNM (genes *DNM2* and *CCDC78*), and autosomal-recessive CNM (genes *BIN1, MAP3K20,* and *SPEG*).

**Figure 1:**
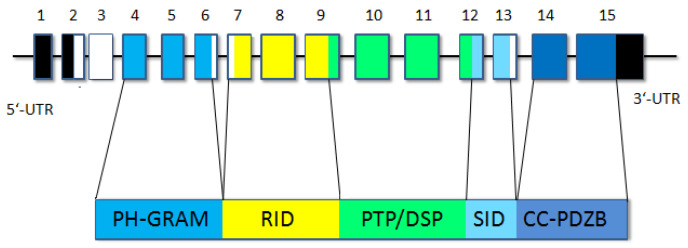
A schematic representation of *MTM1* exons encoding myotubularin domains.

Myotubular myopathy 1 (myopathy, centronuclear, X-linked, OMIM 310400, XLMTM) is one of the most common and severe forms of CNM. Its frequency is estimated to be 1 case per 50,000 male newborns [1]. This form of congenital myopathy is caused by disruptions in the structure of myotubularin, which is a phosphatidylinositol 3-phosphate (PI3P) phosphatase and is essential for the differentiation of muscle cells [1,2,3]. The MTM1 protein consists of 603 amino acids and has 5 domains: PH-GRAM (Pleckstrin Homology-Glucosyltransferase, Rablike GTPase Activator and Myotubularin), RID (Rac1-Induced recruitment Domain), PTP/DSP (protein tyrosine phosphatase/dual-specificity phosphatase), SID (SET-protein Interaction Domain), and C-terminal coiled-coil motif (PDZ binding domain) (Figure 1) [4]. Myotubularin is coded by the *MTM1* gene, which is mapped on the long arm of the X chromosome (Xq28) and consists of 15 exons. To date, according to the data presented in HGMD [5], there are 366 described pathogenic variants in the *MTM1* gene. Among these variants, 32% are missense mutations, 16.7% are deletions, 15% are splice site mutations, 13.1% are nonsense mutations, 10.6% are gross deletions, 8.7% are duplications, 2.5% are indels, 0.8% are gross duplications, and 0.6% are complex rearrangements.

XLMTM has three forms distinguished by the severity of symptoms: mild, moderate, and severe. The disease is characterized by an early age of onset, diffuse weakness, and generalized muscular hypotonia [3]. Other possible symptoms include weakness in sucking and swallowing muscles, ophthalmoplegia, respiratory failure, diaphragmatic eventration, hepatic failure, cryptorchism, and joint contractures. Tendon reflexes are decreased or absent [6]. Muscle biopsy examination shows muscle fibers of reduced size with large vesicular nuclei localized in the center of the cell (normal muscle cell nuclei are peripheral) and positioned in one straight line. The predominance of slow contracting type 1 fibers and the absence of oxidative activity in the periphery of the fibers are also detected [6,7]. High vimentin and desmin immunoreactivity is detected in muscle fibers of an affected individual, whereas in muscles of a healthy full-term newborn, these enzymes will be inactive [6]. Laboratory analysis shows creatine phosphokinase levels within the norm. Electromyography results are usually normal; however, minimal non-specific symptoms may be detected in infancy. Nerve conduction velocity may be decreased, although usually within the norm. The disease predominantly affects males [8]. Female carriers are divided into two groups: manifesting carriers (includes mild (muscle weakness and independent ambulation), moderate (muscle weakness and assisted ambulation), and severe phenotypes (muscle weakness and wheelchair dependence) and non-manifesting carriers (they have no muscle weakness and can be independently ambulant). Non-manifesting carriers may present with clinical symptoms such as fatigue, intolerance to physical activity, myalgia, and cramps. The development of clinical symptoms in manifesting carriers may be related to skewed X chromosomal inactivation [8,9]. At the same time, manifest carriers with a mild phenotype may not seek medical attention, so they may not be diagnosed and will be detected by chance. Manifest carriers with a moderate or severe phenotype may seek medical attention, but they may be diagnosed with a diagnosis other than XLMTM, as XLMTM is not usually suspected in women [9].

## 2. General Characteristics of the Family and Examination Methods

**Figure 2:**
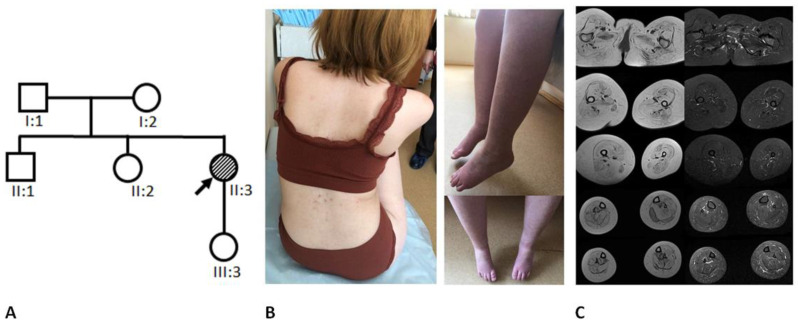
(**A**). The proband’s pedigree. (**B**). The proband’s back and lower limbs: bilateral scapula winging, kyphoscoliosis, diffuse muscle atrophy (**C**). Muscle MRI: symmetrical severe fat replacement of all pelvic girdle and leg muscles, relatively spared medial gastrocnemius muscle on the left side.

We examined a nonconsanguineous family from Moscow Oblast, Russian Federation (family tree is presented in Figure 2A). The proband was examined in the counselling unit of the Research Centre for Medical Genetics at the age of 32 years. The proband’s medical documents and biological materials (blood, fibroblast, and buccal epithelium samples), as well as blood samples of the proband’s daughter, parents, sister, and brother, were provided (it was not possible to obtain a biopsy of the muscle tissue of the proband due to the complexity of the material sampling procedure).

Molecular genetic analysis was carried out on samples of genomic DNA extracted from peripheral blood leukocytes, buccal epithelium cells, and fibroblasts, as well as on samples of RNA extracted from fresh peripheral blood leukocytes. Molecular genetic analysis was carried out using mass parallel sequencing (MPS) on a next-generation Ion S5™ sequencer (Thermo Scientific, Waltham, MA, USA). The probes were prepared using ultramultiplex PCR, followed by sequencing (AmpliSeq™). We used the “congenital muscular dystrophy” target panel (developed based on the Ion Ampliseq technology) including coding sequences of the following genes: *SELENON*, *LMNA*, *TPM3*, *ACTA1*, *PLOD1*, *MYPN*, *ITGA7*, *STAC3*, *CNTN1*, *MYF6*, *CFL2*, *KBTBD13*, *TRIP4*, *CHST14*, *CCDC78*, *MYH2*, *FKRP*, *TNNT1*, *RYR1*, *DNM2*, *COL6A3*, *KLHL41*, *BIN1*, *ZAK*, *SPEG*, *COL5A2*, *COL6A1*, *COL6A2*, *CHKB*, *ITGA9*, *KLHL40*, *LMOD3*, *MTMR14*, *MEGF10*, *LAMA2*, *COL12A1*, *DSE*, *FKBP14*, *AEBP1*, *TPM2*, *FKTN*, *COL5A1*, *MTM1*, and *VMA21*. The read depth of the detected variant in samples of DNA extracted from blood leukocytes exceeded ×164. The detected variant was verified by direct automated Sanger sequencing. The variants were analyzed using the hg19 genomic assembly, data from HGMD Professional v2022.1 [5], and the data interpretation guidelines [10]. The DNA samples were genotyped with PCR using the «AmpFISTR Identifiler Direct PCR Amplification Kit» (Applied Biosystems) for direct amplification of 16 loci of human DNA according to the manufacturer’s protocol, followed by amplification product separation on a 3130 xl Genetic Analyzer (Hitachi, Ibaraki, Japan). The ratio of inactivated X chromosomes was established by analyzing the methylation of the *AR* promotor and *AR* (gene located on the X chromosome) trinucleotide repeat quantity detection using specific primers GCTGTGAAGGTTGCTGTTCCTCAT/AGCGCAGCACCTCCCGGCGC. The X chromosome haplotypes were determined using the Investigator^®^ Argus X-12 QS (Qiagen, Hilden, Germany) kit with subsequent amplification product separation on a 3130 xl Genetic Analyzer (Hitachi). The canonical transcript modification was demonstrated using mRNA analysis. The RNA was extracted from the proband’s peripheral blood leucocytes using the QIAamp^®^ RNA Blood Mini Kit (Qiagen). The quality of total extracted RNA was evaluated using agarose gel electrophoresis and spectrophotometric detection of the 260/280 ratio during the measurement of total RNA concentration. The reverse transcription of total RNA was carried out using the QuantiTect Reverse Transcription Kit (Qiagen). Standard PCR for the proband’s cDNA samples, as well as control samples of healthy people’s cDNA, was carried out using specific primers GAACATATCAAGCTCGTTTTGAC/GTCAAAACAGTTCCCTACAGC.

The detection of the pathogenic variant in the *XIST* gene was carried out using direct automated Sanger sequencing on the 3130 xl Genetic Analyzer (Hitachi). Standard PCR for genomic DNA samples of the proband and her sister was carried out using specific primers GTTCTTTCTGGTATGTCTTTGCTTTC/GTATCGGATACCTGCTGATTCCC.

## 3. Results

### 3.1. Clinical Data

The proband is a 32-year-old, non-ambulant female with complaints of weakness in her arms and legs, a nasal voice, and choking while eating. She has had symptoms since birth. She was a floppy baby with a weak cry and ptosis of the right eyelid. At the age of 6 months, muscle weakness was noted. She had a motor development delay: she started to sit at the age of 8 months and to walk at the age of 19 months. In childhood, she always walked on her toes and could never jump. She attended a special school for children with cerebral palsy. The disease progressed, and at the age of 12 years, the proband switched to home education because of prominent muscle weakness and gait disturbances. After pregnancy and delivery by cesarean section at the age of 20 years, she became non-ambulant. Since then, the condition has been slowly progressing. For several years, she has noticed her voice changing and choking when eating. During in the last two years, after starting physical exercises, she noticed an increased improvement in muscle strength.

The case is first in the family: she has a healthy brother and sister and a healthy daughter (Figure 2A).

At the time of examination at the age of 32 years, her body weight was 44 kg, and her height was 146 cm. The proband has a high-arched palate, midface retrusion, moderate facial weakness, asymmetrical eyelid ptosis, horizontal and vertical right ophthalmoparesis, and horizontal strabismus without diplopia. In addition, dysphonia and dysphagia are noted, but the pharyngeal reflex is normal. Muscles of the upper and lower limbs are diffusely atrophied (Figure 2B). The proband has bilateral scapula winging and kyphoscoliosis, weakness of the neck flexors and extensors (2/5), shoulder girdle muscles (2/5), hand muscles (4/5), and pelvic girdle and leg muscles (0–1/5). Contractures of the knee and ankle joints are noted, and she has hypoplasia in the fourth and fifth toes. Arm tendon reflexes are reduced, patellar reflexes are absent, and ankle reflexes are normal. The proband did not show evidence of respiratory failure or skeletal asymmetry.

The proband’s creatine phosphokinase (CPK) level was normal, measured twice (50 and 70 U/L). Muscle MRI (**Magnetic resonance imaging**) of the lower limbs showed diffuse symmetrical severe fat replacement of all pelvic girdle and leg muscles without signs of inflammation (Figure 2C). The medial gastrocnemius muscle on the left side was relatively spared in comparison with other lower leg muscles.

Considering the data obtained, a preliminary clinical diagnosis of “congenital myopathy” was made and later turned out to be erroneous.

### 3.2. Molecular Genetic Analysis

**Figure 3:**
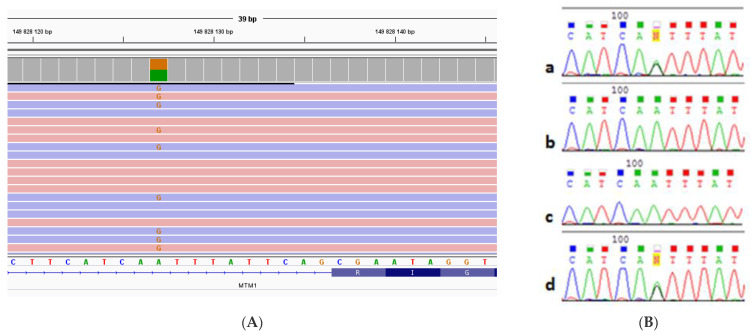
(**A**). The result of the MPS of genomic DNA extracted from peripheral blood leukocytes of the proband. A replacement in the 11th intron of the *MTM1* gene is presented. (**B**). The result obtained in the study of intron 11 of the *MTM1* gene by Sanger sequencing in genomic DNA extracted from peripheral blood leukocytes for: (**a**) the proband, (**b**) the proband’s mother, (**c**) the proband’s father, and (**d**) the proband’s daughter. The c.1261-10A>G nucleotide sequence variant was detected in the proband and her daughter in a heterozygous state.

We analyzed the genes included in the “congenital muscular dystrophy” target panel, which also includes congenital myopathy genes, using MPS. The analysis detected a heterozygous c.1261-10A>G nucleotide sequence variant, previously described as pathogenic [11,12,13], in intron 11 of the *MTM1* gene (NM_000252) (Figure 3A).

According to the literature data and the Splice AI prediction program, the detected variant leads to a disruption in the acceptor splice site of exon 12 and causes the activation of the cryptic site, which leads to a nine-nucleotide elongation of exon 12 [11], thus adding three extra amino acids on the protein level. The read depth of this point was ×164. After that, we searched for the detected variant in genomic DNA samples (extracted from peripheral blood leukocytes) of the proband’s parents, brother, sister, and daughter using Sanger sequencing. The patient’s daughter has the pathogenic c.1261-10A>G variant in intron 11 of the *MTM1* gene (NM_000252) in a heterozygous state. This variant was not detected in the proband’s parents and siblings. The relationship establishment showed both parents to be true biological parents; therefore, the nucleotide sequence variant detected in the proband is de novo (Figure 3B).

**Figure 4:**
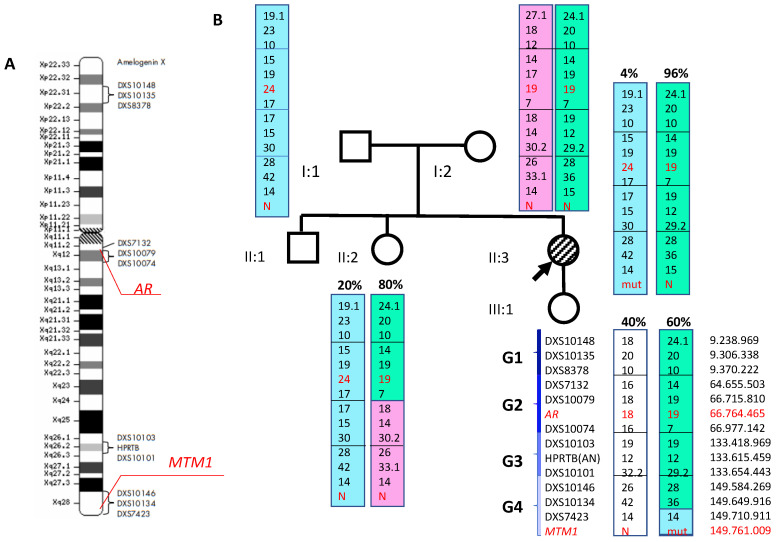
(**A**). A schematic representation of the X chromosome with locations of markers included in the Investigator^®^ Argus X-12 QS kit. (**B**). A schematic representation of the X chromosome haplotypes (genomic DNA isolated from peripheral blood lymphocytes was studied). The proband’s father has 24 CAG repeats in exon 1 of the *AR* gene, while the mother has 19 (highlighted in red). The proband’s maternal X chromosome containing the allele with 19 repeats is inactivated by 96%, while the paternal chromosome containing the allele with 24 repeats is inactivated by 4% and is therefore active. The proband’s sister has the 19-repeat chromosome inactivated by 80%, and the 24-repeat by 20%. The proband’s daughter did not have skewed inactivation. Colors indicate the X chromosome haplotypes in the family. As shown by the presented data, the *MTM1* gene is located below the DXS10134 marker, and as a result of crossover, the X chromosomes exchanged the loci containing the *MTM1* gene.

After that, we determined the methylation pattern in the (CAG)n repeat region in exon 1 of the *AR* gene in samples of genomic DNA (extracted from peripheral blood leukocytes) of the proband, her mother, sister, and daughter, as well as in samples of genomic DNA extracted from the proband’s buccal epithelium cells and fibroblasts. The analysis of genomic DNA extracted from peripheral blood leukocytes showed the skewed X chromosome inactivation in the proband and her sister (proband—96%: 4%, (CAG)n repeats 19/24; the proband’s sister—80%: 20%; (CAG)n repeats 19/24) (Figure 4).

The proband’s daughter did not have skewed X chromosome inactivation (40%: 60%, (CAG)n repeats 18/19). The analysis of the proband’s genomic DNA extracted from buccal epithelium cells and fibroblasts also showed skewed X chromosome inactivation (90%: 10%, (CAG)n repeats 19/24 and 99%: 1%, (CAG)n repeats 19/24, respectively). It was impossible to evaluate the X chromosome inactivation ratio in the proband’s mother because of the presence of CAG repeats in a homozygous state ((CAG)n 19/19). Fragment analysis using the Investigator^®^ Argus X-12 QS kit allowed us to determine the X chromosome haplotypes in samples of genomic DNA of the proband and her parents, sister, and daughter (Figure 4). Haplotyping results showed that the c.1261-10A>G nucleotide sequence variant in the proband’s *MTM1* gene occurred on the paternal X chromosome, which contains allele 24 of the *AR* gene and is not methylated (active). The proband’s daughter inherited the c.1261-10A>G nucleotide sequence variant in the *MTM1* gene from her mother; however, during maternal meiosis, a recombination occurred between X chromosomes. As a result, the daughter’s mutant X chromosome contains allele 19 of the *AR* gene (Figure 4).

**Figure 5:**
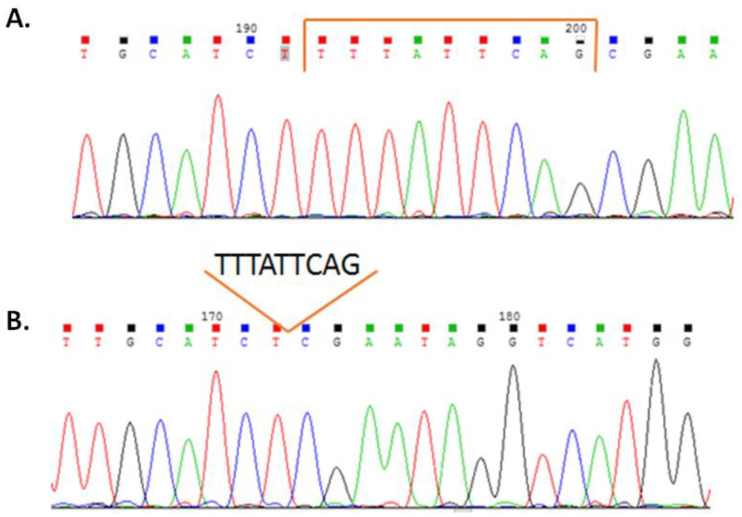
(**A**). Sequencing results for the proband’s *MTM1* mRNA extracted from fresh peripheral blood leukocytes. The bracket indicates the 9-nucleotide insertion. (**B**). Sequencing results for control *MTM1* mRNA. The location of the 9-nucleotide insertion in the elongated transcript is indicated.

The mRNA (extracted from peripheral blood leukocytes) of the proband’s *MTM1* gene was analyzed to determine the canonical transcript modification. Only one *MTM1* transcript with an elongated exon 12 was detected (Figure 5).

Seeing that the skewed X chromosome inactivation was detected in two biological sisters, we decided to search for the most common pathogenic variant located in the promotor of the *XIST* gene, which is causative for skewed inactivation (c.-4C>G, NR_001564.2) [14,15]. Sanger sequencing did not detect this particular variant.

## 4. Discussion

X-linked myotubular myopathy predominantly affects males and is generally characterized by early onset and severe phenotype, often leading to early death [8]. However, there are approximately 40 described cases of female patients with MTM. All of them had symptoms characteristic for congenital myopathy. Examination of muscle tissue biopsy showed signs of centronuclear myopathy [8]. All female patients were carriers of pathogenic variants in the *MTM1* gene. Some of them were the only affected family members; others had male relatives who were affected. Most affected females had skewed X chromosome inactivation [8,16], but in some cases, no skewed inactivation was detected [8,17]. All of them were diagnosed with myotubular myopathy.

With that, to establish the “X-linked myotubular myopathy” diagnosis in a female patient, it is not enough to detect a pathogenic variant in the *MTM1* gene and skewed X chromosome inactivation without *MTM1* mRNA analysis and muscle tissue biopsy examination results. It is necessary to determine that the active chromosome carries the pathogenic variant in the *MTM1* gene.

The detected c.1261-10A>G nucleotide sequence variant was first described as pathogenic by Beatrice M. de Gouyon in 1997. This variant was detected in four male patients with clinical and histological signs of centronuclear myopathy. The analysis of three patients’ mRNA (extracted from peripheral blood lymphocytes) of the *MTM1* gene detected a transcript of this gene with a nine-nucleotide elongation of exon 12, leading to an insertion of three amino acids Phe-Ile-Gln on the protein level [11]. In 1998, Ichizo Nishino also described this variant in a Japanese male patient with clinical and histochemical signs of centronuclear myopathy. mRNA (extracted from muscle tissue) of the *MTM1* gene was also analyzed for this patient [18]. In the following years, this variant was described as pathogenic multiple times [19,20] and is currently considered to be a common pathogenic variant leading to severe CNM in male patients. The c.1261-10A>G variant is localized in intron 11 of the *MTM1* gene. According to the data presented in literature, the Splice AI prediction program, and the mRNA analysis of the proband’s *MTM1* gene carried out in our laboratory, the detected variant leads to a disruption in the acceptor splice site of exon 12 and causes the activation of a cryptic site, which leads to a nine-nucleotide elongation of exon 12 [11] and, therefore, the insertion of three amino acids on the protein level. The insertion occurs in a highly conservative catalytic PTP domain of myotubularin, which presumably affects the catalytic activity of the protein. The c.1261-10A>G nucleotide sequence variant is not registered in the control cohorts of the Genome Aggregation Database (gnomAD), RuExac (the genetic variant database of the Research Centre for Medical Genetics accumulating data of 2090 patients) [21], and RuSeq (unified Russian genetic variant database from the Genetico laboratory, SerbaLab, and Saint-Petersburg’s municipal hospital № 40 accumulating data of 1500 patients) [22]. According to the data presented in HGMD [5], there are 24 described pathogenic variants in the exon 12 *MTM1* gene. Of these, 33.4% (8) are missense mutations, 20.8% (5) are deletions, 16.6% (4) are splice site mutations, 16.6% (4) are nonsense mutations, and 12.6% (3) are duplications. This corresponds with the general distribution of the *MTM1* pathogenic variant. With that, most of the pathogenic variants (both in general for the gene and in exon 12) are missense mutations, which may correlate with the enzyme activity of the myotubularin protein. Out of the 93 described deletions and duplications in the *MTM1* gene, 12 are pathogenic variants not leading to a frameshift. Thus, despite the fact that the c.1261-10A>G nucleotide sequence variant does not lead to a frameshift, it is pathogenic. In this case, considering all the data mentioned above, as well as the fact that this variant is *de novo* and is located on an active paternal X chromosome, we confirmed that the detected c.1261-10A>G variant in intron 11 of the *MTM1* gene is the molecular genetic cause of the disorder in the proband (according to the ACMG criteria—PP3, PM2, PM4, PM1, PS2, PP5).

We confirmed that the skewed X chromosome inactivation led to disease manifestation in a female carrier of a pathogenic variant of the *MTM1* gene. In other studies, different mechanisms of clinical symptom manifestation in female carriers are considered [16,17,23]. The discussion is based on the absence of skewed X chromosome inactivation in female patients in some cases, as well as the absence of correlation between the disease’s severity and the degree of the X chromosome’s inactivation skew. If the authors only determined the inactivation ratio by analyzing the methylation of the *AR* promotor, it is entirely possible that the results (no skew detected) do not represent the actual situation. A. V. Panova states that methylation of the *AR* locus does not correlate with the X chromosome inactivation ratio in female stem cells with induced pluripotency and recommends carrying out clinical diagnostics using alternative regions or locus combinations, which would allow one to analyze the X chromosome inactivation ratio with more precision [24]. The same conclusion was made by B. Bertelsen et al., who determined the differences between X chromosome inactivation ratios obtained with various methods, including *AR* and *FMR1* analysis. The authors also suggest using several loci simultaneously to determine the skew of X chromosome inactivation as a more informative approach [25].

In the current case, the female proband has milder clinical symptoms in comparison to male patients with the same pathogenic variant, which could be caused by the minor expression of a gene located on the X chromosome with 96% inactivation.

Seeing that different tissues from the same patient may have different X chromosome inactivation patterns [26], we obtained samples of different tissues (peripheral blood, fibroblasts, buccal epithelium), with the exception of muscle tissue, from the proband. Considering the fact that skewed inactivation was detected in all analyzed samples, we assumed it to be skewed in the proband’s muscle tissue as well.

The obtained data on the presence of skewed X chromosome inactivation in both sisters indicate its possible hereditary cause. It may be caused by the occurrence of a pathogenic variant in the *XIST* gene, which leads to a non-random active X chromosome choice, thus making the chromosome carrying the mutant *XIST* allele always active [27]. We did not detect the most common pathogenic variant in the promoter of the *XIST* gene; however, this does not exclude this gene being causative for skewed inactivation. It is entirely possible that there is another pathogenic variant affecting the expression of this gene; therefore, sequencing of the whole *XIST* gene might be useful. It is also worth noting that aside from *XIST*, there are other genes involved in the X chromosome inactivation process, such as *TSIX*.

This case shows that even in the age of mass parallel sequencing, traditional molecular genetic analysis methods are not outdated; on the contrary, they play a new and significant role as instruments for the verification of variants detected with MPS.

## Data Availability

Not applicable.
